# *Burkholderia cepacia* complex in cystic fibrosis: critical gaps in diagnosis and therapy

**DOI:** 10.1080/07853890.2024.2307503

**Published:** 2024-01-23

**Authors:** Juan Carlos Gutiérrez Santana, Victor Rafael Coria Jiménez

**Affiliations:** Laboratory of Experimental Bacteriology, National Institute of Pediatrics, Mexico City, Mexico

**Keywords:** *Burkholderia cepacia* complex, cystic fibrosis, antimicrobial stewardship, early diagnosis, antibacterial agents

## Abstract

*Burkholderia cepacia* complex (*Bcc*) is a bacterial group with ‘natural’ multi-antimicrobial resistance. This complex has generated epidemic outbreaks across the world. In people with cystic fibrosis (CF), *Bcc* can cause severe lung infections that lead to accelerated lung damage, which can be complicated by necrotizing pneumonia accompanied by high fevers, leucocytosis, and bacteraemia, which commonly causes fatal outcomes. Specifically, infection by *Burkholderia cenocepacia* is considered an exclusion criterion for lung transplantation. The species of *Bcc* exhibit both genetic and phenotypic hypervariability that complicate their accurate microbiological identification. Automated methods such as MALDI-TOF can err in the determination of species. Their slow growth even in selective agars and the absence of international consensuses on the optimal conditions for their isolation make early diagnosis a difficult challenge to overcome. The absence of correlations between antibiograms and clinical results has resulted in the absence of standardized cut-off values of antimicrobial susceptibility, a fact that brings a latent risk since incorrect antibiotic therapy can induce the selection of more aggressive variants that worsen the clinical picture of the host, added to the absence of a clear therapeutic guide for the eradication of pulmonary infections by *Bcc* in patients with CF, resulting in frequently ineffective treatments. There is an urgent need to standardize methods and diagnostic tools that would allow an early and accurate diagnosis, as well as to perform clinical studies of the effectiveness of available antibiotics to eradicate *Bcc* infections, which would allow us to establish standardized therapeutic schemes for *Bcc*-infected patients.

## *Burkholderia cepacia* complex

The genus *Burkholderia* was discovered in 1949 by the American bacteriologist Burkholder while analysing the skin of rotten onion fragments. As of 2016, this extensive group of β-proteobacteria consistsed of approximately 70 species [1] and has undergone multiple reclassifications over the years, coupled with the integration of new taxa [2], so by September 2023, the genus had already reached a total of 130 taxa, as published in the List of Prokaryotic Names with Standing in Nomenclature (LPSN, https://lpsn.dsmz.de/genus/burkholderia).

The species of this genus have high phenotypic diversity and are ubiquitously distributed in the environment [1–3]. They are found in diverse ecological niches that include water, soil, industrial environments, artificial products, plants, and invertebrate and vertebrate animals [3–5] They even manage to frequently colonize cosmetic, disinfectant, and pharmaceutical products meant for hospital use [6]. Their genome is 6–10 Mbp^4^ and is made up of at least two chromosomes [4, 7] and a diverse combination of plasmids [4]. Some specimens of certain species, such as *Burkholderia cenocepacia* (*B. cenocepacia*), have even exhibited up to three chromosomes and a plasmid (strain J2315) [1].

The genus *Burkholderia* is commonly divided into two large groups known as the *Burkholderia pseudomallei* complex (*Bpc*) and *Burkholderia cepacia* complex (*Bcc*) [1]. This manuscript describes the ability of *Bcc* members to generate infectious outbreaks, the challenges for the early and accurate diagnosis of infections caused by these bacteria, specifically in people suffering from cystic fibrosis (CF), and the absence of eradication therapies standardized for this population.

## *Bcc* as an opportunistic pathogen

The set of bacteria grouped under *Bcc* has shown remarkable characteristics, such as their ability to biodegrade materials [8], primarily xenobiotics [7, 9], a fact that has positioned them as important microorganisms in agriculture to promote plant growth [4, 8], control the emergence and spread of pathogens for crops (biocontrol), and assist in soil bioremediation [4, 7, 9]. Their participation in areas that are socioeconomically necessary for humans has made human–bacteria interactions inevitable [7].

They are highly infectious, causing infections in anyone under any circumstance [8]. Although they primarily affect immunocompromised individuals, since the 1950s they have acquired the classification of a human pathogen under the name *Pseudomonas cepacia* [6, 7].

## Outbreaks of nosocomial infections by *Bcc*

Between 1971 and 2019, a total of 111 infectious outbreaks associated with *Bcc* were reported in different hospitals, affecting 2390 patients on different continents and causing a total of 240 deaths [10, 11]. *Burkholderia* is considered one of the main causal agents of sepsis [11] commonly associated with various health care products [6].

Among the recently reported outbreaks, we can mention that which took place in February 2018 in the USA, 60 patients treated in different hospitals in California, New Jersey, Pennsylvania, Maine, Nevada, and Ohio were infected with *Bcc*. The infections were associated with the use of batches of no-rinse cleaning foam contaminated with these microorganisms, as determined by the similarity between a clinical isolate and isolates of samples of the pharmaceutical product identified by pulsed-field gel electrophoresis (PFGE). However, the failure to register the batches used in the patients prevented the exact determination of the batches specifically contaminated with the variants found in the patients [6].

Between June and July 2018 in Chesapeake, Virginia, USA, *Bcc*-positive blood cultures were identified in three patients who had previously undergone ultrasound-guided intravenous catheterization. Notably, more comfortable, used ultrasound gel containers had been refilled with another product that came in a less comfortable dispenser. PFGE typing distinguished the similarity between the clinical isolates and the gel cans used, showing the relevance of not reusing medical material in the clinical management of patients [12].

In Germany between August and September 2018, by means of whole-genome sequencing (WGS), lung infections caused by the same strain of *Bcc* were identified, which coincided with isolated specimens of two closed oral rinse vials (MWS) containing octenidine [13], a substance recommended in that country to prevent ventilator-associated pneumonia [14], that were of a lot used from three weeks before the outbreak. Two of the three infected patients died nine to 10 days after the identification of the microorganism, despite being treated with antimicrobials [13]. Because of this, Bender J. et al. (2022) undertook a national surveillance project of *Bcc* infections associated with exposure to contaminated MWS, identifying 36 cases from six hospitals located in four federal states of Germany. Four cases were retrospectively identified 5 months before the outbreak described by Becker S. et al. (2018) [13], while the last finding was identified in November of that same year. A total of five patients died manifesting atypical pneumonia and additional conditions specific to each individual. Methodologically, random batches of MWS from the manufacturer were selected. By PFGE and WGS, they identified similarity between 14 isolates from 12 patients treated in three different hospitals, those isolated from closed cans of MWS, and those from three patients previously studied by Becker et al. (2018). In parallel, 11 isolates from 10 patients treated in another hospital coincided with a different batch of MWS, while the isolates from the other 14 patients treated in other hospitals did not show molecular coincidence with the microorganisms isolated from the batches of MWS analysed, suggesting the likelihood that these batches have not been sampled [14].

In February 2019, an outbreak of *Bcc* was identified in the paediatric unit of a university hospital in Turkey, affecting six patients between 8 months and 14.5 years of age in a period of 7 days. Typing by PFGE showed molecularly identical strains in these patients. This hospital suspended admission of new patients. Treatment was based on the results of the antibiogram, which showed susceptibility to trimethoprim-sulfamethoxazole (SXT), and once negative cultures for *Bcc* were obtained, admission of patients resumed [15].

In March 2019, an infectious outbreak by *Bcc* was identified in four patients treated in a medical-surgical intensive care unit in Turkey, which increased to six patients by the month of April. PFGE typing revealed similarity (>90%) between the clinical isolates of the patients, so a search was undertaken for the reservoir. It turned out to be a batch of MWS with chlorhexidine that had been used in the clinical management of the patients. Five of the six patients died despite the antibiotic therapies provided, although the retrospective analysis ruled out that the cause of death of one of the patients was *Bcc* infection [16].

Shortly before the COVID-19 pandemic, an outbreak of *Bcc* was identified in four dialyzed patients at Queen Mary Hospital, Hong Kong, China, in September 2019. The patients had manifested at their dialysis exit ports in the form of serous to bloody secretions. The reservoir was identified in aqueous chlorhexidine solutions that had been contaminated during their dilution process at the manufacturing sites of two different brands. Thus, the retrospective investigation of *Bcc* infections in patients treated between 2014 and 2019 distinguished a substantial increase in infections from March 2018, while the typification by multilocus sequence typing (MLST) and WGS determined that the infections had been caused by microorganisms of two different phylogenetic groups that had contaminated antiseptic solutions made by two respective manufacturers [17].

The outbreaks described show the importance of a continuous examination of hospital environments, as well as of the products used for health care, including those products intended for disinfection [6]. Furthermore, the combination of available diagnostic and epidemiological tools is essential to distinguish *Bcc* infections in a timely manner, allowing an appropriate clinical approach to affected individuals. However, the constant search for and notification of cases of nosocomial infections due to *Bcc* are not mandatory practices in different cities and nations [6, 16], which contributes to the constant risk for people with certain predisposing characteristics, such as those suffering from chronic obstructive pulmonary disease (COPD), ventilator‑associated pneumonia (VAP) [18], or CF [1, 5, 19].

Some authors have recognised that the infection caused by this pathogen is difficult to eradicate due to its high intrinsic resistance to many antibiotics [18]. In fact, lung infections caused by these bacterial species exhibit significantly more severe clinical manifestations in these patients [1, 5, 19]. Specifically, in CF is characterized by very rapid lung damage [7, 20] that drastically shortens life expectancy [20], coupled with the fact that these hosts are usually the reservoir of ‘epidemic’ strains that have been associated with a mortality risk up to 3.92–5 times greater than that caused by other *Bcc* variants, such as the ET-12 strain [21, 22].

## CF and constant lung infections

CF is a rare and fatal monogenic disease [5, 23–25] commonly affecting Caucasian individuals of European descent [26–28]. They have an approximate life expectancy of 40 years in developed countries [5], while in underdeveloped nations the maximum life expectancy is 19 years [29].

This genetic disorder, caused by mutations in the CF transmembrane conductance regulator gene (*CFTR*) [25, 30, 31], is characterized by low volume of paraciliary fluids in the pulmonary epithelia [32], which affects the composition and viscosity of mucus [33], giving it an extremely thick, dehydrated [26, 33, 34], and sticky nature [26, 34] that favours the establishment of lung infections by diverse bacterial species, such as *Pseudomonas aeruginosa* (*P. aeruginosa*), *Staphylococcus aureus*, *Haemophilus influenzae*, *Stenotrophomonas maltophilia*, *Achromobacter* xylosoxidans, and *Bcc* [35, 36], the latter being the group of pathogens considered the most virulent and threatening among CF patients [36].

## *Bcc* in CF

The *Bcc* complex is made up of at least 22 bacterial species of the genus *Bulkholderia* [20, 21, 37], which for reasons still unknown have a ‘preference’ for colonizing the lungs of CF patients [8]. Its incidence and prevalence are low in the CF population, with an annual incidence of 7.4% in some nations, such as Canada [22], and a global prevalence of 0.07–5.00% [2, 3, 7, 20], which has been decreasing due to the implementation of the astringent measures to which these patients are commonly subjected [2, 7, 38].

## Lung infection by *Bcc* in patients with CF

Despite preventive measures, pulmonary infections due to *Bcc* continue to appear sporadically in the population with CF^2^. Characterization by MLST has established that individuals with this condition commonly acquire the infection from diverse natural environments [2, 7]. A high transmissibility of *Bcc* has been observed in these individuals [4, 9, 39] in both clinical and community settings [40], and it can be transmitted even by simple social contact [4].

Among the *Bcc* species usually identified in cultures of respiratory secretions of people with CF are *B. cenocepacia*, *Burkholderia stabilis*, *Burkholderia multivorans* (*B. multivorans*), and *Burkholderia vietnamensis* [1, 7], the latter considered the species most susceptible to aminoglycosides [7]. Unfortunately, 85–97% of lung infections caused by *Bcc* in this population worldwide are by *B. cenocepacia* and *B. multivorans* [1, 20, 40, 41], which are recognized as the most virulent species of this complex [7, 21], 94% and 50% of the respective infections being chronic despite the aggressive antibiotic therapies applied [42–45].

The clinical manifestations of *Bcc* infection in people with CF are highly variable and unpredictable [4, 45], although they are often associated with a more severe and rapid lung impact than that caused by other colonizing bacteria in the airways of CF patients, such as *P. aeruginosa* [2, 22, 24, 38]. *Bcc* commonly cause exacerbated lung inflammation that dramatically worsens the lung function of CF patients [2, 22, 24]. In addition, they can cross the epithelial barrier, enter the bloodstream, and lead to bacteremia [45]. *B. cenocepacia* has been associated with a mortality rate up to 5 times higher than that of other *Bcc* species and constitutes an exclusion criterion for lung transplantation [7, 20] due to the high risk of death it brings to patients after this surgical intervention [2, 22, 39].

## Chronicity of *Bcc* infection and clinical complications

Like *P. aeruginosa* in CF, *Bcc* can express a mucoid colonial morphology derived from the production of exopolysaccharides (EPS) in response to the different selective pressures to which they are subjected in the airways of these hosts [4]. The appearance of the mucoid phenotype indicates an intermediate course of infection where the strains acquire a plethora of changes in different aspects, such as their metabolism, mobility, biofilm formation, and virulence [2]. Therefore, the emergence of these variants adds complexity to the enormous phenotypic diversity *per se* of this bacterial group.

Although the mucoid colonial phenotype of *Bcc* has been associated with less lung damage, it favours its persistence in the airways of infected patients [4]. The nonmucoid variants induce a rapid and severe loss of lung function [2, 4, 42]. *In vitro* experiments have shown that some antibiotics commonly used in the clinical management of CF patients with respiratory infections, such as ciprofloxacin [42] and ceftazidime (CAZ) [4], can induce changes in the mucoid phenotype to the nonmucoid phenotype [42], so incorrect treatment could favour phenotypic changes in the infecting strain, directly worsening the lung damage.

One of the rare but most serious complications of *Bcc* infection in CF patients is known as cepacia syndrome (CS) [1, 4, 20, 21]. A clinical manifestation that some authors have suggested could be related to the presence of severe pathogenic variants of *CFTR* [21], which can manifest itself approximately five years after the first isolation of *Bcc* [21], evidencing the ability of these bacteria to persist in the lung environment despite various selective pressures.

CS is characterized as a sharp, necrotizing pneumonia [1, 20, 21] that is accompanied by high fevers, bacteremia [20, 21], and leukocytosis [7, 22] causing a rapid decline in lung function [20, 21] and thus a poor prognosis [1, 20], with a fatal outcome in approximately 50% of cases [20, 21].

## Microbiological identification of *Bcc*

**Figure 1.**
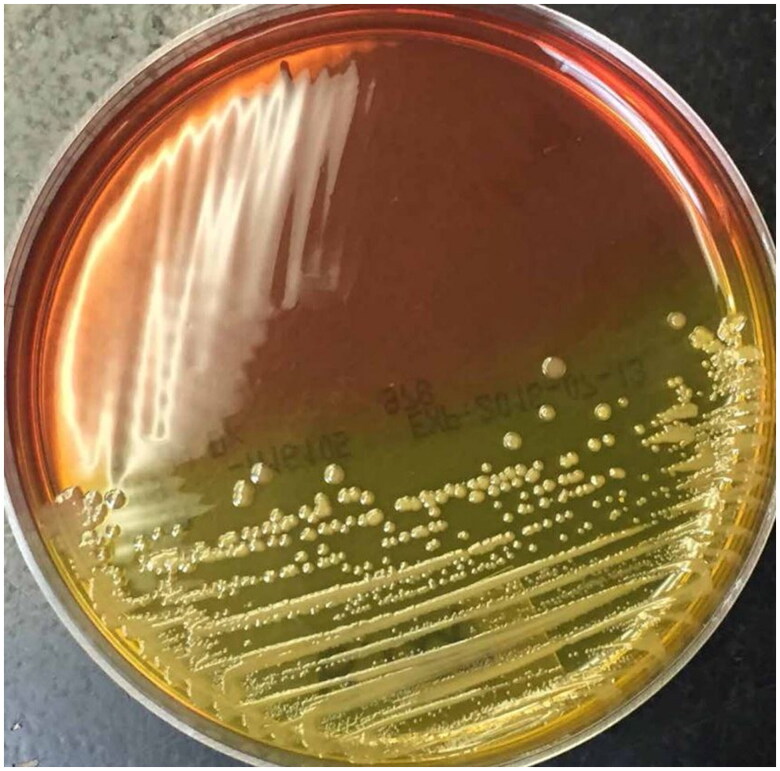
*Bcc* growth and colonial isolation on BCSA medium. BCSA agar plate seeded by cross streaking with a Bcc strain showing yellow colour in the section with bacterial growth (Taken from Landes N. et al. 2016 under the terms and conditions of licence number 5687200897100 (ELSEVIER)).

The microbiological diagnosis of *Bcc* infection in individuals with CF has been recognized as complex [13, 14, 46]. Therefore, early diagnosis [14, 45, 47, 48] is a difficult goal to achieve due to various factors, such as the slow growth of *Bcc* even in selective media (48 to 72 h) [7, 39]. These media include the selective agar for *B. cepacia* (BCSA) (Figure 1) [49], *Pseudomonas cepacia* agar (PCA), and the oxidation medium fermentation of polymyxin, bacitracin, lactose (OFPBL), and there is no consensus on the optimal conditions for their isolation [39, 50].

Additionally, the determination of the genus and species by biochemical tests is difficult and often leads to incorrect results [14, 15] due to the close genetic relationship between each of the members that make up *Bcc,* which is visualized as a high diversity of very similar phenotypes [14, 46]. Even commercial bacterial identification systems [46, 50] as well as automated equipment such as MALDI-TOF devices have shown deficiencies in distinguishing the different members of this complex to the species level [7, 17, 46, 50, 51], which can have significant clinical implications [46].

For these reasons, the diagnosis is usually complemented by molecular tests, particularly PCR amplification of the genetic region of *recA* [7, 8, 14, 37]. The identification based on the sequence of 16S rRNA has shown too little genetic variation to differentiate between all the species of *Bcc* [14, 46] since it exhibits a similarity of >97.7% between the members of this bacterial group^50^. Some authors even suggest a multiple molecular diagnosis consisting of the sequencing and analysis of the genetic targets *gyrB* [52, 53], *recA*, 16S rRNA, *hisA*, and *rspU* [50], in addition to genotyping by MLST, which has been essential to identify and classify strains previously misclassified, as well as for the local and global epidemiological evaluation of *Bcc* [2, 7, 14, 46].

## Determination of the antimicrobial susceptibility of *Bcc*

One of the essential items in microbiological diagnosis is the determination of antimicrobial susceptibility, since an appropriate treatment based on antibiograms reduces the likelihood of selecting variants that are increasingly resistant to antimicrobials [7, 37, 48, 54]. Even the unnecessary use of antibiotics, their incorrect administration, or an insufficient concentration accelerates the global growth of the crisis of antimicrobial resistance [48, 55, 56]. However, for *Bcc,* there is a shortage of standardized international criteria for the interpretation of antibiograms [7, 37, 40, 46].

The large number of belonging to *Bcc* has complicated the establishment of cut-off values for the available antimicrobial agents because each species can exhibit a significantly different susceptibility profile [37, 40]. In addition, its interpretation can vary depending on the observer even following the same standard procedure [40]. The disc diffusion method provides low reproducibility for *Bcc* members [7]. Therefore, more expensive or time-consuming techniques, such as the E-test or the broth dilution and agar dilution methods, have been suggested [7, 37, 40, 57].

The Clinical and Laboratory Standards Institute, USA (CLSI) has released cut-off values (http://em100.edaptivedocs.net/dashboard.aspx) for the minimum inhibitory concentration (MIC) of ticarcillin-clavulanate, CAZ, meropenem (MEM), minocycline (MI), levofloxacin, trimethoprim-sulfamethoxazole (SXT) and chloramphenicol, while the cut-off values for the disk diffusion method are only available for CAZ, MEM, MI and SXT. In contrast, the European Committee on Antimicrobial Susceptibility Testing (EUCAST) has not set cut-off points for any antibiotic under either method (https://mic.eucast.org/search/) (Table 1), mainly due to the few studies that exist on antimicrobial susceptibility in this complex [40], as well as the absence of correlations between the MICs visualized *in vitro* and the clinical results observed in patients [7, 19, 37, 40].

**Table 1.** Updated antibiogram published by EUCAST until december 2023 (modified from https://mic.eucast.org/search/).

Antibiotic	MIC (µg·mL^-1^)																			Observations	Confidence Interval
	0.002	0.004	0.008	0.016	0.03	0.06	0.125	0.025	0.5	1	2	4	8	16	32	64	128	256	512		
FPZ	0	0	0	0	0	0	0	2	1	4	34	32	28	8	2	2	0	0	0	113	ID
CAZ	0	0	0	0	0	0	0	1	5	13	20	9	5	2	8	3	4	1	0	71	0.25-256
CTB	0	0	0	0	0	0	0	0	1	2	6	3	0	0	0	1	0	2	0	15	ID
LEV	0	0	0	0	0	0	0	0	0	4	9	3	0	1	0	0	0	0	0	17	ID
MEM	0	0	0	0	0	1	2	8	4	12	23	17	4	5	0	5	0	0	0	81	ID
MXF	0	0	0	0	0	1	2	5	2	0	1	2	0	0	2	0	0	0	0	15	ID
TZP	0	0	0	1	0	0	1	6	18	12	6	6	5	3	2	3	5	1	1	70	0.125-16
TEM	0	0	0	0	0	0	0	0	0	3	12	30	43	32	17	3	6	1	0	147	2-1024
TM	0	0	0	0	0	0	1	0	1	0	6	2	2	11	4	4	6	3	0	40	ID
SXT	0	0	0	0	0	0	0	4	4	2	5	2	0	0	0	0	0	0	0	17	ID

FPZ = Cefepime-zidebactam; CAZ = Ceftazidime; CTB = Ceftibuten; LEV = Levofloxacin; MEM = Meropenem; MXF = Moxifloxacin; TZP = Piperacillin-tazobactam; TEM = Temocillin; TM = Tobramycin; SXT = Trimethoprim-sulfametoxazole; ID = Insufficient data.

## Suggested antimicrobials for the eradication of *Bcc* in CF patients

Antibiotic therapy is essential in the clinical management of CF patients infected by *Bcc* [20] since timely eradication can prevent the rapid decline of lung function and improve quality of life [41]. Studies in the Cochrane database of systematic reviews have recognized that therapy for *Bcc* lung infections in people with CF is a significant challenge [42, 58, 59], due to factors as diverse as the natural multi-antimicrobial resistance of *Bcc* species [1, 2, 40], their intrinsic resistance to disinfectants and antiseptics [16], their strong ability to develop resistance to other antimicrobial agents [1, 3, 20], including those of last option [60], the high variability in the expression of virulence factors in each strain [45], their ability to survive intracellularly in lung epithelial cells and macrophages [21, 45], and the establishment of biofilms [7]. Thus, the choice of the appropriate antibiotics is complicated due to the innate resistance of *Bcc* to carboxypenicillins, first-second-generation cephalosporins, polymyxins [7] and according to EUCAST Guidance Document on *Burkholderia cepacia* complex, to all aminoglycosides (https://www.eucast.org/eucastguidancedocuments). There are reports of *in vitro* studies on the reduced antimicrobial activity of aminoglycosides such as amikacin and tobramycin (TM) on these microorganisms [37], as well as the inability of rifampicin to inhibit the establishment of *Bcc* biofilms, even in combination with miconazole, econazole, or ketoconazole [7].

The few *in vitro* tests on antimicrobial activity have suggested SXT, CAZ, chloramphenicol, MI, imipenem, MEM, doripenem, ceftolozane-tazobactam, ceftazidime-avibactam, and some fluoroquinolones, such as ciprofloxacin [1, 7, 19, 37, 46], coupled with cefiderocol (FDC) [20], as the hypothetically most effective treatments against these bacterial species [1, 19, 50] since *Bcc* exhibit susceptibility to these antibiotics [20, 37]. Recently, it has been reported that a clay mineral from Kisameet Bay in Canada, known as Kisameet clay, demonstrated in *in vitro* tests of Kisameet clay solutions at 10% (w/v) and an incubation time of 24 h, the ability to reduce and eliminate the viability of 10 strains of *Bcc*, except for *B. multivorans* and *Burkholderia dolosa* (*B. dolosa*), which species needed 48 h of incubation with the solution [36].

## Antibiotic therapy for the eradication of *Bcc* in patients with CF

Despite the information provided by the disseminated antibiograms and the findings of *in vitro* studies, there is still no consensus or guideline on the therapeutic regimens applicable to people with CF infected by *Bcc* [3, 42, 45, 58, 59].

So far, only one randomized clinical study has evaluated prolonged antibiotic therapy commonly applied to CF patients [31, 61, 62]. This was carried out in health care centres in the USA over 52 weeks and included 100 people with CF between 6 and 57 years old. It not found differences in mortality, quality of life, and sputum density of 52 patients treated with 75 mg of inhaled aztreonam three times a day for 24 weeks, compared to 48 individuals who received placebo [58, 59].

The scarcity of clinical studies has led to antibiotic therapies being chosen primarily because of the imprecise results of antibiograms [7, 19, 20], the experience of the treating specialist [7], or the findings of the few case series and case reports [41], while the duration of therapy is commonly based on the clinical and microbiological response visualized [50]. Empirically, aggressive treatments with different combinations of antibiotics have been suggested [5, 41, 42, 58–60], complemented by different routes of administration, such as nebulized application [5, 41, 60] or inhalation [41, 42, 58], since this route of administration permits a concentration up to 100 times higher than that of the antimicrobial agent [58], coupled with the possibility of being administered in conjunction with oral and intravenous antibiotics, which would hypothetically delay or prevent the establishment of chronic infections [41, 42].

Reports on the application of different treatment regimens have shown that inhaled administration of TM is insufficient to suppress pulmonary infection by *Bcc* in patients with CF^7^. Therefore, its synergy with other compounds has been explored, such as amiloride or verapamil, compounds that have demonstrated *in vitro* the ability to inhibit efflux pumps [7] and block sodium channels [41], which have managed to increase the antimicrobial activity of TM [7, 41]. The clinical results of these combined treatments have shown effectiveness in most cases [7, 41, 63]. However, infections caused by *B. dolosa* [7] and *B. cenocepacia* [63] have exhibited notorious difficulties [7, 63]. This same therapeutic scheme supplemented with oral cotrimoxazole allowed the eradication of infections caused by various species of *Bcc* except for those established by *B. multivorans* [41], which are some of the most virulent species in this complex.

On the other hand, the combined use of TM with FDC in a patient with CF who presented episodes of pulmonary exacerbations by *B. cenocepacia* every 2 months allowed researchers to extend the period of absence of exacerbations up to 5 months, although the patient still died [20]. Likewise, in a host with CF infected by *B. multivorans* subjected to multiple treatments with up to seven antibiotics, including FDC, it was not possible to improve the deteriorated clinical status of the patient [60].

These reports show a weak correlation between the antimicrobial activity identified *in vitro* and the clinical results seen in the patients subjected to the treatments [7, 45]. Not only antibiotics but also other antimicrobial agents, such as bacteriophages, have shown promising results in *in vitro* studies, although clinically they have shown difficulties clearing out of *Bcc* lung infections, so they have not been able to prevent death due to lung deterioration of hosts [60]. Furthermore, *Bcc* infections in CF patients resist the 10 most commonly used antibiotics in the clinic [2]. This leads to inefficient frequent eradication treatments [40].

It is evident that we need to carry out exhaustive research on the antimicrobial activity of various compounds through *in vitro* and *in vivo* tests with the more than 22 species that make up the *Bcc* complex, which would allow us to define standardized MIC values to evaluate them through clinical studies of therapeutic combinations in infected individuals and would allow the establishment of standardized therapeutic regimens [7, 20, 41] and in the improvement of the lung function of the infected patient, as well as the reduction in their morbidity and mortality, thereby improving their life expectancy and quality of life [42]. Complementing such efforts would be the fulfilment of the request by the World Health Organization (WHO) to carry out research and development of novel antimicrobial agents to fight against multi-antimicrobial-resistant pathogens [48, 64, 65], such as *Bcc* [37].

## Conclusion

The species of *Bcc* exhibit an intrinsic resistance that allows them to persist in the presence of antiseptics, disinfectants, and antibiotics, translating into a potential latent risk that individuals with certain diseases and predispositions, such as CF, will contract *Bcc* infections that will lead to severe pulmonary dysfunction and a high risk of mortality. There is a need for constant epidemiological surveillance of equipment and products destined for private, cosmetic, and medical use.

The early and accurate microbiological diagnosis of pulmonary infection by *Bcc* in people with CF is complicated by the lack of international consensus on the optimal conditions for their isolation, since the species of this complex present a high diversity of phenotypes and genotypes that commonly result in misidentification.

The use of sequencing to analyse more than one genetic region would allow the identification of the species to greater accuracy but would significantly increase the cost of the diagnosis, making it inaccessible for various laboratories, since the tools used in molecular epidemiology are also expensive, though they are essential in clinical management to identify the reservoir and take the necessary epidemic measures.

Additionally, the methods suggested to analyse the antimicrobial susceptibility of isolates are expensive, and there is currently no clear correlation between the inhibitory activity of different antibiotics and the clinical results. The available case reports demonstrate the therapeutic success of different treatment schemes, although they often indicate deficiencies against the most virulent species of the complex, suggesting the existence of additional resistance mechanisms that allow it to overcome the selective stress of aggressive treatments.

There is a notorious lack of clinical studies clearly indicating the effectiveness of any therapeutic strategies. Treatment with the wrong antibiotic can induce the emergence of more aggressive bacterial variants that complicate the clinical picture of the host.

It is urgent to carry out intense research on the determination of the antimicrobial activity of various compounds through *in vitro* and *in vivo* tests, specifically in CF models, and their application in randomized clinical trials to find standardized therapeutic regimens that will improve the lung function of the infected patient, eradicate the pathogen, reduce the morbidity and mortality of the hosts, and thereby improve their quality of life and prolong their life expectancy, not to mention contributing to the constant fight against the crisis of antimicrobial resistance.

## Data Availability

The data presented in this review were taken from published literature on public domains and therefore data sharing is not applicable to this article, as no new data were created or analysed in this study.
